# Hypopharyngeal Perforation Following Foreign Body Ingestion: A Case Report

**DOI:** 10.7759/cureus.19708

**Published:** 2021-11-18

**Authors:** Raghad K Alsalamah, Abdulaziz K Alaraifi, Abdulaziz A Alsalem, Khurram Waheed

**Affiliations:** 1 Medicine, King Saud Bin Abdulaziz University for Health Sciences, Riyadh, SAU; 2 Otolaryngology-Head and Neck Surgery, King Abdulaziz Medical City, Ministry of National Guard Health Affairs, Riyadh, SAU

**Keywords:** aerodigestive tract, otorhinolaryngology, foreign bodies, perforation, hypopharynx

## Abstract

Foreign body ingestion is a common complaint frequently seen in otolaryngology. Some sharp foreign bodies may get impacted in the aerodigestive tract causing a perforation. However, hypopharyngeal perforation is a rare injury that needs early recognition due to its significant morbidity. In this case report, we report a case of hypopharyngeal perforation caused by foreign body ingestion in an adult patient. A 60-year-old female presented with a foreign body sensation in the throat, dysphagia, and odynophagia. A neck CT scan showed a foreign body in the hypopharynx with a collection of free air along the posterior pharyngeal wall. She underwent laryngoscopy and esophagoscopy for examination and foreign body removal. Following the procedure, the patient was treated conservatively for a week and then discharged home in a stable condition. Hypopharyngeal perforation following foreign body ingestion is uncommon. A high index of suspicion is required to reach an early diagnosis and treatment.

## Introduction

Upper aerodigestive tract foreign body is a common emergency complaint frequently seen in otolaryngology [1]. Foreign bodies aspiration or ingestion can occur in all age groups, and they are easily removed from both the airway and the esophagus using bronchoscopy or esophagoscopy, respectively [2]. However, some sharp foreign bodies may get impacted in the aerodigestive tract causing a perforation [3].

Hypopharyngeal perforation is a rare injury that needs early recognition, which if missed, can result in significant morbidity [4]. Because of the septic contents and the continuous movements during swallowing and breathing, hypopharyngeal perforation can lead to serious complications such as mediastinitis, pleural empyema, sepsis, and even death [5]. Hypopharyngeal perforation can result from a variety of causes, including penetrating injury or blunt trauma to the neck or chest [6]. However, hypopharyngeal perforation caused by a foreign body is rarely discussed and published in the literature. We present a case of hypopharyngeal perforation caused by foreign body ingestion that was treated conservatively.

## Case presentation

**Figure 1:**
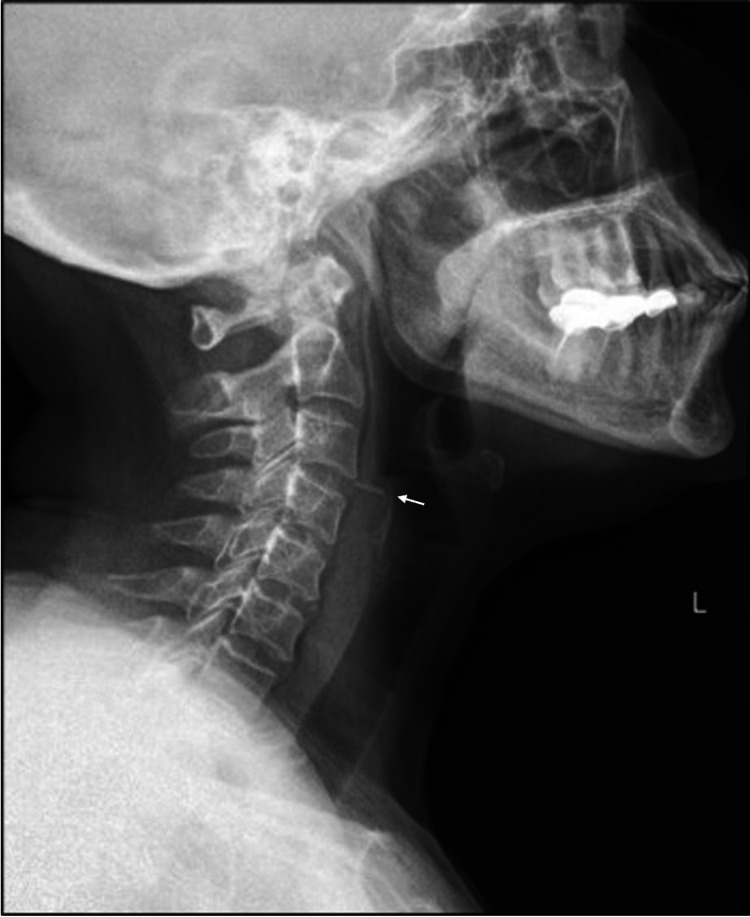
A lateral neck soft tissue x-ray showing a foreign body (arrow) in the hypopharynx with a linear radiolucency in the retropharyngeal space representing free air

**Figure 2:**
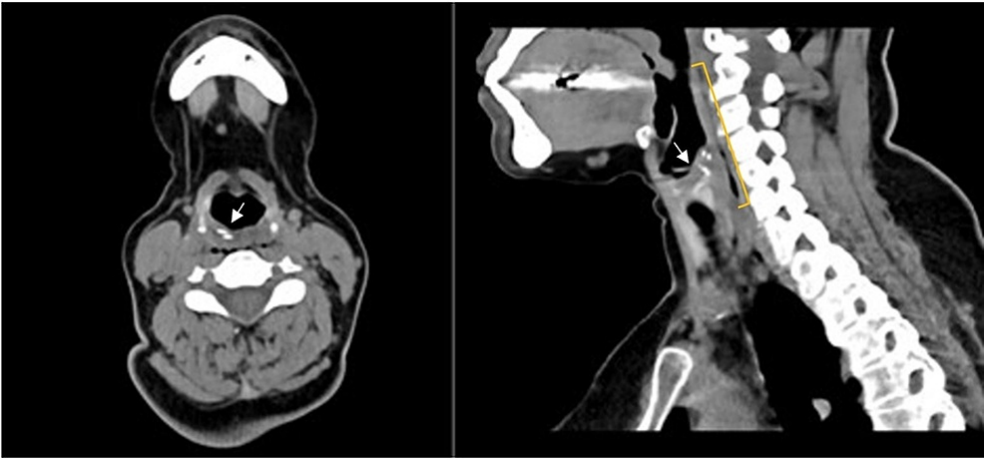
A neck CT scan showing a linear hyperdense foreign body in the hypopharynx (arrows) with a collection of air along the retropharyngeal space (right bracket) representing a concealed perforation

A 60-year-old female was referred from the emergency department with a foreign body sensation in the throat, dysphagia, and odynophagia. The symptoms developed while the patient was having her lunch. She had no other related gastrointestinal complaints (i.e., hematemesis, drooling, or vomiting) or airway-related complaints (i.e., choking, cyanosis, cough, dyspnea, or hemoptysis). The patient’s medical and surgical histories were only significant for bronchial asthma, dyslipidemia, fibromyalgia, and hypertension. She was hemodynamically stable and saturating well on room air with no signs of respiratory distress. Physical examination was unremarkable except for mild tenderness over the anterior neck above the level of the thyroid cartilage. A lateral neck soft tissue X-ray confirmed the presence of a foreign body in the hypopharynx with a linear radiolucency in the retropharyngeal space representing free air (Figure 1). A neck computed tomography (CT) scan demonstrated a linear hyperdense foreign body in the hypopharynx with free air along the retropharyngeal space representing a concealed perforation (Figure 2).

**Figure 3:**
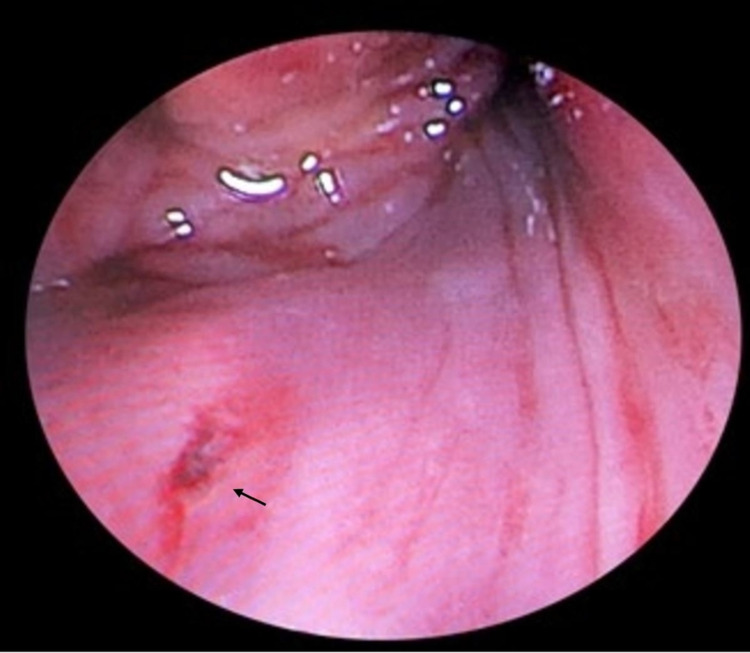
Endoscopic view showing a small wound in the posterior pharyngeal wall representing the site of the perforation (arrow)

The patient was admitted for endoscopic examination under general anesthesia and foreign body removal. The patient was intubated using a flexible fiberoptic bronchoscope to avoid dislodging the foreign body. During fiberoptic intubation, a whitish plastic foreign body was found in the hypopharynx, which was removed using foreign body forceps under endoscopic guidance. Examination post foreign body removal showed a small wound in the posterior pharyngeal wall, which represents the site of the perforation (Figure 3). The examination was completed using rigid esophagoscopy, which showed a normal intact esophagus. Following the procedure, the patient was treated conservatively with close observation, strict nasogastric tube (NGT) feeding, analgesics, and intravenous piperacillin/tazobactam 4.5g every 8 hours for seven days. An upper gastrointestinal series using Gastrografin (Bayer, Leverkusen, Germany) was performed after a week of the incident and showed a normal contrast passage through the hypopharynx and esophagus without any evidence of leakage. Flexible nasopharyngeal endoscopy was repeated and demonstrated intact nasopharynx, oropharynx, with a small and almost healed posterior pharyngeal wall perforation. The patient was allowed to resume oral feeding and was discharged home in a stable condition.

## Discussion

Hypopharyngeal perforation is a rare injury that needs early recognition due to its serious complications. Delayed diagnosis can result in significant morbidity and potential mortality [4]. It is usually seen with penetrating injury to the neck or chest [7]. However, it might also result from iatrogenic causes such as diagnostic or therapeutic endoscopy and endotracheal intubation. Iatrogenic perforation occurs most often in emergencies during manipulations by less experienced physicians [8,9]. External blunt trauma is another cause representing less than 2% of hypopharyngeal perforation cases [6]. Foreign body ingestion resulting in hypopharyngeal perforation however is a rare cause. To the best of our knowledge, only a few cases of hypopharyngeal perforation caused by foreign body ingestion have been reported in the literature [3,10,11]. The majority of cases of hypopharyngeal perforation due to foreign body ingestion have reported a fishbone as the causative object, unlike our patient who was found to have a sharp plastic object in the hypopharynx [10]. Fishbones are among the most common foreign bodies reported in the upper aerodigestive tract [12].

Patients with hypopharyngeal perforation may present with subcutaneous emphysema that extends proximally and distally, chest or neck pain, odynophagia, hoarseness, stridor, or hemoptysis [13]. Sore throat, dysphagia, and pyrexia are late symptoms of the injury. Pyrexia is often associated with retropharyngeal abscess formation, which can lead to carotid artery pseudoaneurysm formation if left untreated. Missed or delayed diagnosis is associated with serious complications include mediastinitis, pleural empyema, septic shock, and death [4]. Our patient only had dysphagia, odynophagia, and mild neck tenderness with no significant complications.

There is no clear diagnostic approach for hypopharyngeal perforation in the literature. Clinical signs and symptoms along with endoscopic assessment are sufficient to consider a definitive diagnosis in some cases [14]. Plain radiograph, CT, and contrast swallow scans can be used in combination, if there is clinical suspicion of the diagnosis [6]. Neck and chest radiographs are necessary for the initial evaluation of the upper aerodigestive tract foreign bodies and may reveal the presence of cervical and mediastinal emphysema [7,15]. CT scan can detect small air collection and is used preoperatively to plan an appropriate surgical approach as it shows the exact size and extent of the injury [7,16]. Our patient had a neck CT scan that showed a foreign body in the hypopharynx with a small collection of air along the posterior pharyngeal wall. However, the standard evaluation for the upper aerodigestive tract is direct visualization [17]. Endoscopy helps acquire information about the presence, site, and extent of the perforation as well as inspects the involved area for pharyngeal edema or hematoma [17]. Our patient underwent laryngoscopy and esophagoscopy, which showed a foreign body in the hypopharynx with a minor wound in the posterior pharyngeal wall representing the site of the perforation.

Because of its rarity, the management of hypopharyngeal perforation remains controversial. The management decision depends on many factors, including the size and location of the perforation, the patient's hemodynamic status, and the presence or absence of complications [18]. Published studies recommend conservative management in patients who are hemodynamically stable with a perforation less than 2 cm in size [18]. The management consist of intravenous broad-spectrum antibiotics, parenteral nutrition, and NGT feeding [18]. Surgical intervention is recommended in cases with systemic toxicity, perforations greater than 2 cm, extension to the esophagus, and penetrating injuries [18]. Our patient was managed conservatively as she had a small perforation with no evidence of complications or any involvement of the esophagus. Patients should be monitored for several days for possible complications, and follow-up imaging studies can be done depending on the general condition of the patient [19]. Our patient underwent an upper gastrointestinal study and nasopharyngeal endoscopy a week after conservative management, which showed no sequelae of injury as a result of foreign body.

## Conclusions

Hypopharyngeal perforation following foreign body ingestion is uncommon. A high index of suspicion is required to achieve an early diagnosis and treatment. The management can be either conservative or surgical, depending on the clinical findings. Watchful patient monitoring during the treatment period is recommended to achieve better outcomes and avoid serious complications.
